# Linking signaling pathways to transcriptional programs in breast cancer

**DOI:** 10.1101/gr.173039.114

**Published:** 2014-11

**Authors:** Hatice U. Osmanbeyoglu, Raphael Pelossof, Jacqueline F. Bromberg, Christina S. Leslie

**Affiliations:** 1 Computational Biology Program, Memorial Sloan Kettering Cancer Center, New York, New York 10065, USA;; 2 Department of Medicine, Memorial Sloan Kettering Cancer Center and Weill Cornell Medical College, New York, New York 10065, USA

## Abstract

Cancer cells acquire genetic and epigenetic alterations that often lead to dysregulation of oncogenic signal transduction pathways, which in turn alters downstream transcriptional programs. Numerous methods attempt to deduce aberrant signaling pathways in tumors from mRNA data alone, but these pathway analysis approaches remain qualitative and imprecise. In this study, we present a statistical method to link upstream signaling to downstream transcriptional response by exploiting reverse phase protein array (RPPA) and mRNA expression data in The Cancer Genome Atlas (TCGA) breast cancer project. Formally, we use an algorithm called *affinity regression* to learn an interaction matrix between upstream signal transduction proteins and downstream transcription factors (TFs) that explains target gene expression. The trained model can then predict the TF activity, given a tumor sample’s protein expression profile, or infer the signaling protein activity, given a tumor sample’s gene expression profile. Breast cancers are comprised of molecularly distinct subtypes that respond differently to pathway-targeted therapies. We trained our model on the TCGA breast cancer data set and identified subtype-specific and common TF regulators of gene expression. We then used the trained tumor model to predict signaling protein activity in a panel of breast cancer cell lines for which gene expression and drug response data was available. Correlations between inferred protein activities and drug responses in breast cancer cell lines grouped several drugs that are clinically used in combination. Finally, inferred protein activity predicted the clinical outcome within the METABRIC Luminal A cohort, identifying high- and low-risk patient groups within this heterogeneous subtype.

Cancers arise through the accumulation of genetic and epigenetic alterations that often target signal transduction pathways, leading to dysregulation of downstream transcriptional effectors and widespread gene expression changes. Since many targeted therapies are small molecule inhibitors of signal transduction proteins or monoclonal antibodies against growth factor receptors, deciphering the signaling pathways that are deregulated in a given tumor in order to personalize therapy is a major goal of cancer genomics. Indeed, large-scale cancer genomics projects have devoted much effort to cataloging somatic alterations across large sets of tumors and mapping them to cellular pathways (The International Cancer Genome Consortium 2010; The Cancer Genome Atlas Network 2012; Curtis et al. 2012). At the same time, these projects have generated massive repositories of tumor mRNA data, giving a complex readout of the transcriptional changes downstream from altered signaling pathways. Nevertheless, we are unable to translate the mutational landscape of a tumor into a usable model of affected pathways, and we are not generally able to use mutational status to accurately predict response to targeted therapies (Baselga 2011). Moreover, despite 10 years of development of pathway analysis approaches (for review, see Khatri et al. 2012), existing tools for associating altered or enriched pathways to mRNA expression profiles give mainly qualitative and noisy results.

The advent of proteomic methods has the potential to provide a systematic map of critical signaling pathways that are altered in cancer. Reverse-phase protein microarrays (RPPAs) are a medium-throughput technology to analyze the expression levels of a protein or phosphoprotein across many samples at once (Paweletz et al. 2001). Recently, The Cancer Genome Atlas (TCGA) project added RPPA profiling for a panel of proteins and phosphoproteins as an additional assay for hundreds of tumors across multiple tumor types. Nevertheless, quantitative profiling of proteins in tumor tissues using RPPA presents many technical challenges, including antibody validation, variability in tissue handling, and intra-tumoral heterogeneity, giving rise to noisy measurements of the activity of signaling proteins. In this work, we hypothesize that we can best extract meaningful information about deregulated signal transduction pathways from RPPA data by linking upstream signaling with downstream transcriptional response, measured by mRNA data, via the transcriptional circuitry. Our model views RPPA data as a noisy readout of the activity of signaling pathways; oncogenic signaling pathways converge on a set of transcription factors (TFs), whose dysregulated activity in turn alters the mRNA expression levels of TF target genes. Formally, we use an algorithm we recently developed, called *affinity regression* (R Pelossof, I Singh, J Yang, M Weirauch, T Hughes, and C Leslie, unpubl.), to learn an interaction matrix between upstream signal transduction proteins and downstream TFs that predicts target gene expression. We use TF binding site prediction to determine the set of TFs that potentially regulate each gene. The trained affinity regression model can then infer the TF activity given a tumor sample’s protein expression profile or infer the signaling protein activity given a tumor sample’s gene expression profile.

We applied our approach to 397 breast cancer profiles from TCGA for which both RPPA and mRNA data are available, using a subset of 192 tumors for training the model. Breast cancer is a heterogeneous disease with diverse pathological features and survival outcomes (Sorlie et al. 2003) and has been categorized into three basic therapeutic groups: (1) basal-like or triple-negative breast cancers (TNBCs, lacking expression of the estrogen receptor [ER], progesterone receptor [PR], and HER2), characterized by a poor prognosis and no specific targeted therapies; (2) HER2 (ERBB2) amplified, associated with relatively poor prognosis if untreated and with significant clinical benefit from anti-HER2-therapy; and (3) estrogen receptor-positive (luminal), characterized by a relatively good prognosis and response to targeted hormonal therapies. Within the ER+ category, gene expression profiling studies (PAM50) have identified at least two subtypes within ER-positive breast cancers, Luminal A and Luminal B (Parker et al. 2009). Although patients with Luminal A cancers have the best prognosis, these tumors are heterogeneous, and there exist few markers that predict recurrence and survival. We used affinity regression to infer the deregulated signaling pathways that drive expression changes in distinct breast cancer subtypes, to leverage the tumor model to predict drug sensitivity using breast cancer cell line mRNA and drug response data, and finally, to predict survival within the heterogeneous ER+, Luminal A subtype. These results provide a detailed case study for how integrative computational analysis can lead to mechanistic and clinically relevant insights into the dysregulated signaling pathways and TFs that underlie differences in cancer subtypes, response to therapy, and clinical outcome.

## Results

### Affinity regression learns an interaction model for signal transduction proteins and TFs

**Figure 1.**
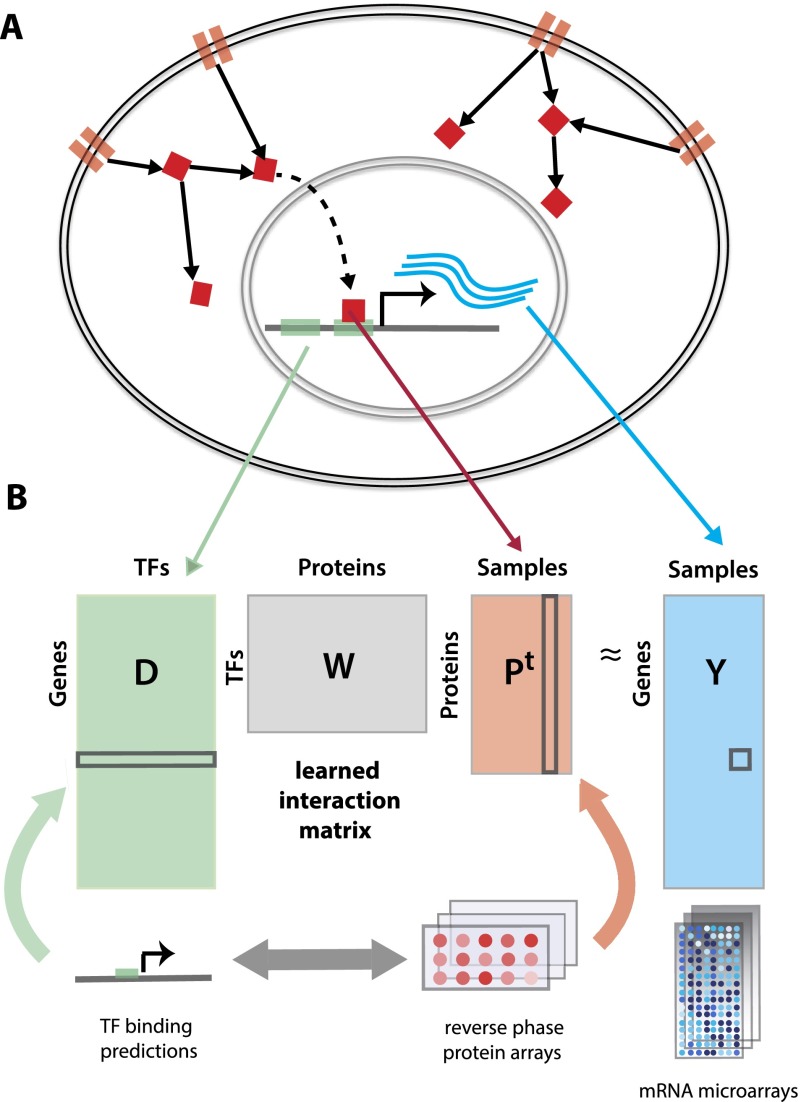
Modeling gene expression variation across tumor samples connects upstream signaling with transcriptional responses. (*A*) The model learns an interaction matrix between upstream signal transduction proteins and downstream TFs that explains target gene expression. (*B*) TF binding site predictions for each gene and RPPA profiles of tumor samples are used to predict gene expression variation relative to a mean tumor expression profile.

Given a set of genes and their gene expression profiles, we use *affinity regression* (R Pelossof, I Singh, J Yang, M Weirauch, T Hughes, and C Leslie, unpubl.) to learn an interaction matrix between signal transduction proteins and TFs that explains target gene expression (Fig. 1). For a data set of *M* tumor samples profiled by microarray across *N* genes, we let *Y* 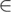 *R*^*N*x*M*^ be the mean-centered log gene expression profiles for all tumor samples, where each column of *Y* corresponds to a microarray experiment. Using TF binding site prediction in gene promoters (see Methods), we define a matrix *D* 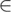 *R*^*N*x*Q*^, where each row represents a gene and each column is a binary vector representing the target genes of a TF. Finally, we define a matrix *P* 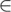 *R*^*M*x*S*^ of tumor sample (phospho) protein attributes where each row represents a tumor sample and each column represents RPPA expression levels of a signaling protein across tumor samples (again mean-centered across RPPA samples). We set up a bilinear regression problem to learn an interaction matrix *W* 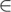 *R*^*Q*x*S*^ for pairs of TF-signaling protein features that predicts target gene expression:
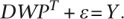


To reduce the dimensionality, we subjected the feature matrix *P* to singular value decomposition prior to training and reduced to a smaller system of equations where the output is the set of pairwise similarities *Y*^T^*Y* between examples rather than *Y* itself (see Methods). Then we used ridge regression to solve for the interaction matrix (for details, see Supplemental Methods; R Pelossof, I Singh, J Yang, M Weirauch, T Hughes, and C Leslie, unpubl.). Since the model captures relationships between signaling proteins, TFs, and gene expression, we can use the trained *W* to obtain different views of a tumor data set: to infer the TF activities in each sample, we can right-multiply the protein expression profiles through the model by *WP*^T^; To infer protein activities in each sample, we can left-multiply the gene expression profile and motif-hit matrix through the model by *Y*^*T*^*DW.* We refer to these operations as “mappings” onto the TF space and the protein space, respectively.

### Affinity regression outperforms nearest neighbor for gene expression prediction on held-out samples

**Figure 2.**
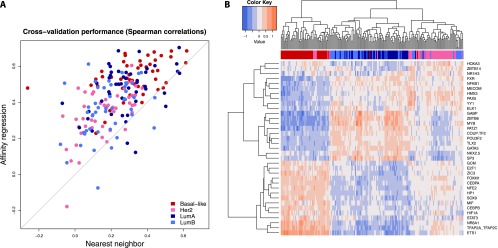
Affinity regression accurately predicts relative gene expression on held-out TCGA breast cancer samples. (*A*) Plot showing Spearman correlations between predicted and actual gene expression changes relative to a median reference using the affinity regression model (*y*-axis) and nearest neighbor (*x*-axis) for TCGA samples representing four breast cancer subtypes (Basal-like, HER2, LumA, LumB). (*B*) Unsupervised hierarchical clustering of tumors based on inferred TF activities recovers Basal-like, HER2, and Luminal (LumA and LumB) subtypes. The clustering was performed using all TFs (see Supplemental Fig. S3), but for readability, only the features with the largest standard deviation across samples are shown in the heatmap.

We evaluated our approach on a data set of BRCA tumors from TCGA where both genome-wide mRNA expression data and RPPA measurements for 164 proteins/phosphoproteins are available. We trained our model on equal numbers of samples for each subtype (*n* = 48 × 4). As motif data, we used binding site predictions for 230 TFs in the promoter regions ([−2kb, 2kb] around the transcription start site) from MSigDB (Liberzon et al. 2011). For statistical evaluation, we computed the mean Spearman rank correlation between predicted and measured gene expression profiles on held-out samples using sixfold cross-validation. We compared our results with a *nearest neighbor* approach, where neighbors are chosen based on similarity of protein expression profiles (input space) as shown in Figure 2A. We obtained a 0.41 (±0.02) mean Spearman correlation between predicted and measured gene expression, compared to 0.23 (±0.02) for nearest neighbor. In contrast, if we randomized motif hits for each gene and RPPA profiles for each tumor, we obtain a Spearman correlation of 0.006 (±0.077). To further validate the performance, we also examined an independent test set of 205 TCGA samples. We obtained a mean Spearman correlation of 0.39 between predicted and measured gene expression, compared to 0.209 for nearest neighbor, similar to the performance difference obtained through cross-validation (see Supplemental Fig. S1). In addition, we evaluated our approach using a newer BRCA RPPA data set from The Cancer Proteome Atlas (TCPA) (Li et al. 2013) and attained similar performance (Supplemental Fig. S2).

Next, we examined whether our model reflects the existing PAM50 expression-based breast cancer subtype classifications (Parker et al. 2009). To identify active TFs for each tumor sample, we mapped its protein expression profile *P*^*T*^ through our learned interaction matrix by *WP*^*T*^ to obtain a weight vector over TFs; here, all training examples (*n* = 192) were used to learn the model. Hierarchical clustering of inferred TF activity of tumor samples (*WP*^*T*^) largely recovered the distinction between the three major subtypes (basal, luminal, HER2), as shown in Figure 2B and Supplemental Figure S3 (adjusted Rand index 0.615 for three-way clustering). In particular, basal-like samples were well separated from other subtypes. However, Luminal A and Luminal B, which are subgroups of the ER-positive subtype, were not as well separated from each other (adjusted Rand index 0.449 for four-way clustering). Clustering was also consistent with ER, PR, and HER2 clinical status (Supplemental Fig. S3).

Similarly, to identify the activity of signaling proteins for each tumor sample, we mapped the expression profiles through the motif hit matrix and our learned model by *Y*^*T*^*DW*. This gives a weight vector over (phospho) proteins for each sample. Clustering the samples by inferred protein activity (*Y*^*T*^*DW*) also recovered the distinction between subtypes, as shown in Supplemental Figure S4 (adjusted Rand index 0.58 for three-way clustering, 0.435 for four-way clustering), in contrast to just using the RPPA values alone (adjusted Rand index 0.289 for four-way clustering) (Supplemental Fig. S5).

These results demonstrate that (1) our affinity regression model explains a meaningful part of the dysregulation of gene expression in breast cancer based on the ability to predict gene expression variation across tumors on held-out tumor samples; and (2) the model largely captures previously defined transcriptomic subtypes.

### Affinity regression identifies subtype-specific TFs and signaling proteins associated with expression changes

Next, we assessed TF-subtype associations using a Mann-Whitney *U*-test to compare inferred TF activity between pairs of transcriptional subtypes or groups of subtypes (see Methods). We tested three pairwise comparisons for each TF: (1) basal-like vs. HER2, Luminal A, Luminal B; (2) HER2 vs. Luminal A, Luminal B; and (3) Luminal A vs. Luminal B. Results of the TF-subtype association analysis are shown in Table 1. (Fewer associations were found using TF mRNA expression levels directly; see Supplemental Table S1.) Basal-like-specific TF regulators include ETS1, a transcriptional regulator implicated in cell development, cell differentiation, cell proliferation, apoptosis, and tissue remodeling (Lincoln and Bove 2005) that has previously been linked to the development of a basal-like breast cancer phenotype (Span et al. 2002; Mylona et al. 2006; Switzer et al. 2012); CEBPB, which has been associated with tumor progression, poor prognosis, and ER-negative status of breast cancers (Milde-Langosch et al. 2003; Zahnow 2009) and whose elevated mRNA expression is associated with metastatic breast cancer (van de Vijver et al. 2002); NFATC4, a member of the nuclear factor of activated T cells (NFAT) family of transcription factors that is involved in immune cell signaling, survival, and angiogenesis (Mancini and Toker 2009) and has been associated with breast cancer cell invasion in ER-negative breast cancer cell lines (Yiu and Toker 2006); high-mobility group (HMG) proteins, nonhistone nuclear proteins known as “architectural transcription factors” that are involved in the regulation of DNA-dependent processes such as transcription, replication, recombination, and DNA repair (Bustin and Reeves 1996; Churchill et al. 1999), have been found in abundance in various cancers types including breast (Peluso and Chiappetta 2010), and include the protein HMGA1, which has been shown to promote metastatic processes in basal-like breast cancer cell lines (Pegoraro et al. 2013); and SOX9, which plays critical roles in development, differentiation, and lineage commitment and whose levels are elevated in a wide range of human cancers including breast (Chakravarty et al. 2011a,b; Matheu et al. 2012).

**Table 1.**
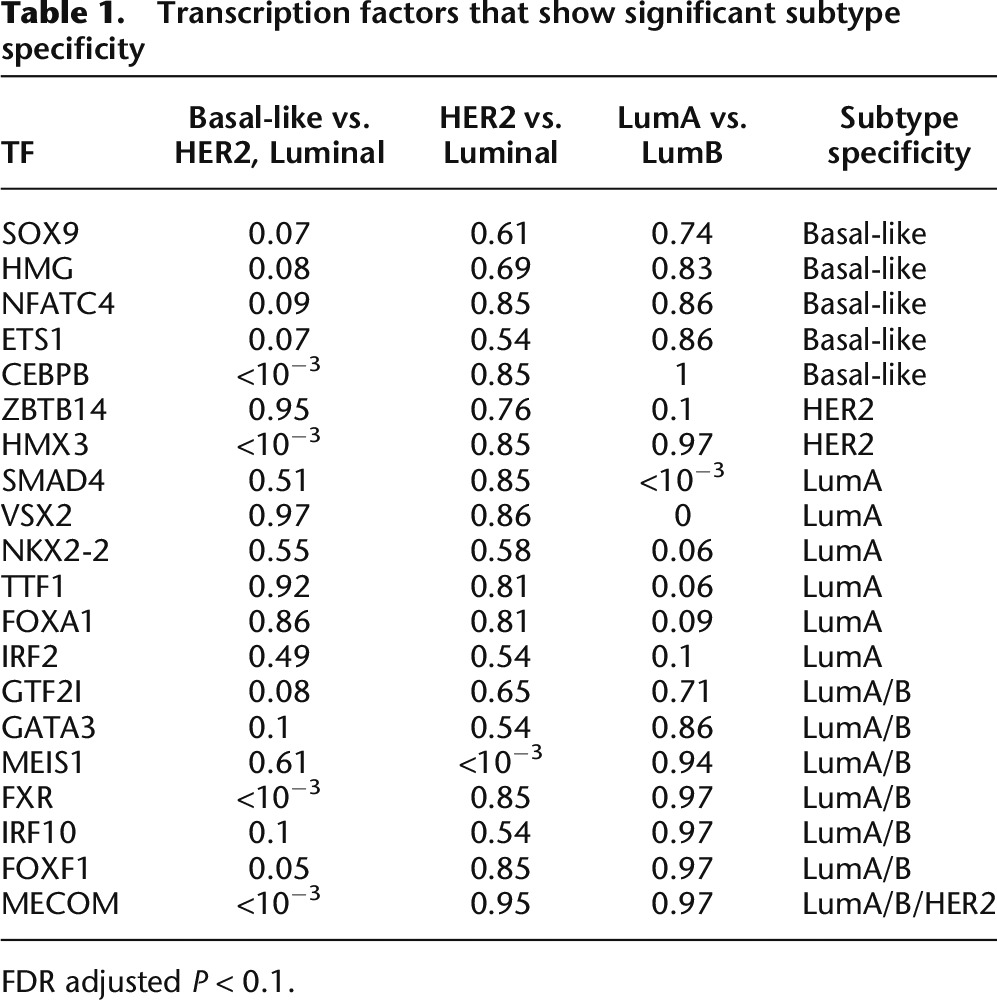
Transcription factors that show significant subtype specificity

TF associations for the other subtypes include HMX3, an ER coactivator, which is inferred to be a HER2-specific TF regulator. *HMX3* has been shown to integrate ESR1 and HER2 receptor tyrosine kinase signaling to promote aromatase expression and hormone resistance in a preclinical model of luminal breast cancer (Cortez et al. 2012). Some of the Luminal A-specific TF regulators include *SMAD4*, which was shown to induce apoptosis in ERα-positive breast cancer cells (Li et al. 2005); NKX2-1 (also known as TTF-1), which regulates genes in the thyroid, lungs, and diencephalon during embryogenesis and whose expression has been detected in a small proportion of breast carcinomas (Robens et al. 2010); and FOXA1, which has been studied within the ERα pathway in luminal breast cancers and found to correlate with patient survival (Badve et al. 2007). One of the TF regulators shared among both the Luminal A and Luminal B groups is *GATA3,* a regulator of ERα signaling that is required for the luminal type of breast cancer (Wilson and Giguere 2008; Dydensborg et al. 2009).

Next, we assessed differences in inferred protein activity across the clinically relevant transcriptional subtypes, again using Wilcoxon rank sum tests in three pairwise comparisons (see Methods): (1) basal-like vs. HER2, Luminal A, Luminal B; (2) HER2 vs. Luminal A, Luminal B; and (3) Luminal A vs. Luminal B. Results are shown in Table 2 (again, fewer associations are found using protein expression levels directly) (see Supplemental Table S2). Briefly, basal-like tumors are associated with higher activity of proteins that have roles in cell cycle progression and proliferation including RB1, CHEK2, CCNE1, MSH6, CTNNB1, and CCNB1. Other signaling proteins included KIT, a transmembrane receptor tyrosine kinase, which was recently proposed as a poor prognostic marker in basal-like breast cancer (Kashiwagi et al. 2013). As expected, the HER2 subtype was associated with higher inferred activity of ERBB2 (pY1248), also known as HER2/NEU. Conversely, PGR (Cancello et al. 2013; Prat et al. 2013), PDK1 (Gagliardi et al. 2012), and PEA15 are associated with Luminal A. Interestingly, in triple-negative breast cancer and ovarian cell lines, increasing PEA15 levels was shown to have an antitumor effect (Bartholomeusz et al. 2006, 2010). Finally, the identified protein signatures for both Luminal A and Luminal B include ESR1, BCL2 (Kim et al. 2012), GATA3 (Kouros-Mehr et al. 2008), INPP4B (Fedele et al. 2010), FN1, CAV1, and CCND1.

**Table 2.**
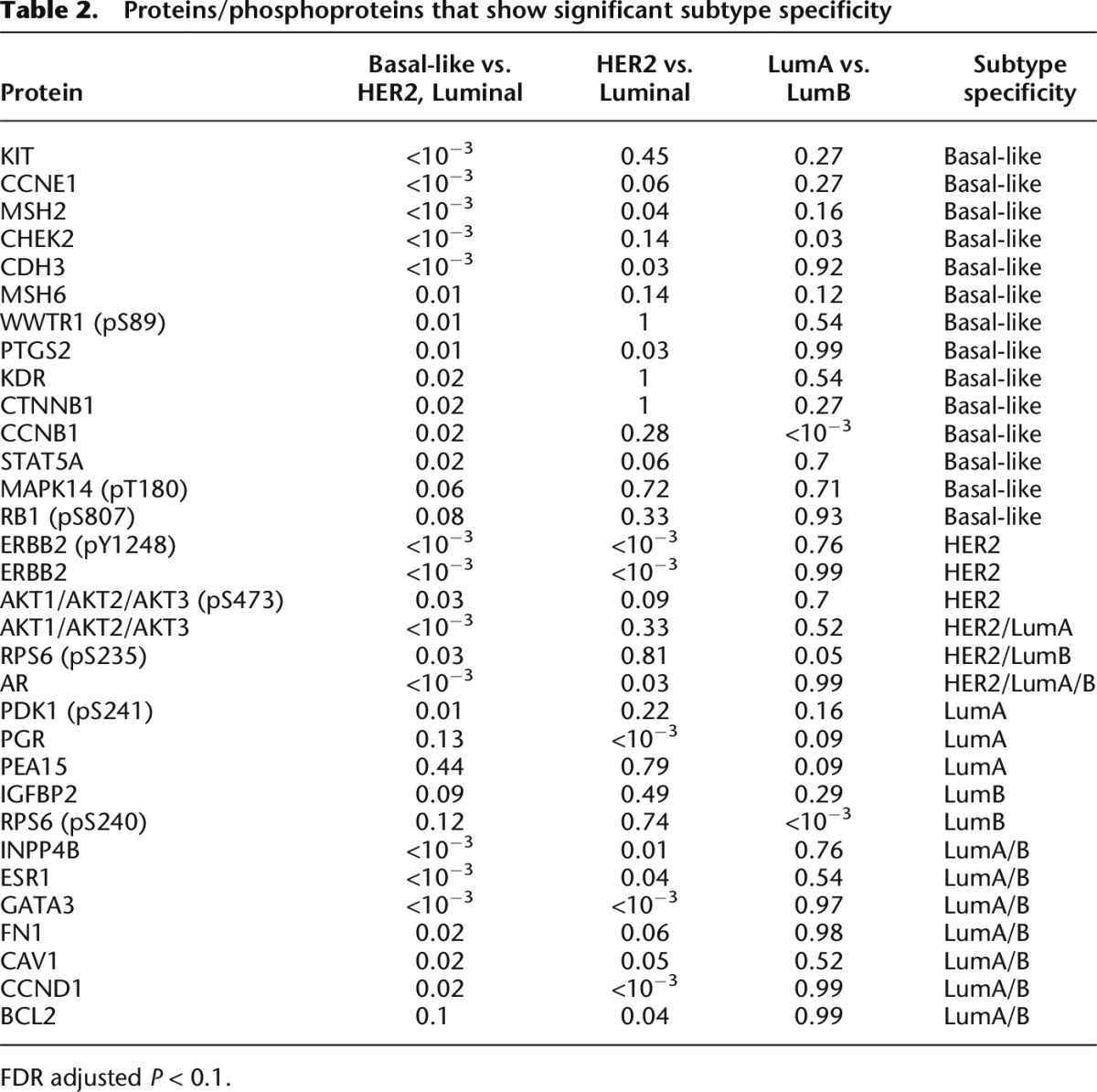
Proteins/phosphoproteins that show significant subtype specificity

When phosphoprotein data is available for a TF, the model may find that its inferred protein level correlates with inferred TF activity (as for GATA3) (Supplemental Fig. S6) or that its protein level is relatively uninformative, although its TF activity varies across samples (e.g., FOXO3) (Supplemental Fig. S7); results for all TFs are listed in Supplemental Table S3.

### Inferred protein activity in breast cancer cell lines can be used to predict drug response

Targeting the pathways that promote growth and invasion of cancer cells is critical for effective treatment of breast cancer. A potentially important application of our approach is through the administration of targeted therapies based on the signaling status of a given patient’s tumor. To address the preclinical feasibility of such an approach, we asked whether our affinity regression model―trained on paired mRNA and RPPA data from breast cancer tumors―could be used to infer protein signaling activity in breast cancer cell lines from their mRNA expression profiles alone, and whether these inferred protein signatures were useful for predicting drug sensitivity. We used previously published gene expression data for 35 breast cancer cell lines (Neve et al. 2006) with corresponding drug response data for 77 drugs quantified by growth inhibition (GI50) (Heiser et al. 2012). The cell lines showed a broad range of responses to most therapeutic compounds. We found that 45 out of 74 (61%) of the drugs produced variable responses across the cell lines (standard deviation of log-transformed GI50 across cell lines greater than 0.5), and we restricted our analysis to these drugs. Out of 45 cell lines, 28 were luminal (ER+), and 15 of those were ERBB2-amplified.

We first used the TCGA-trained affinity regression model to infer protein activity profiles for individual cell lines (*Y*^*T*^*DW*), applied unsupervised hierarchical clustering to these profiles, and confirmed that this clustering discriminated between basal-like and luminal subtypes for the breast cancer cell lines (Supplemental Fig. S8). In contrast, mapping the cell lines through randomized versions of the interaction matrix *W* did not correctly recover basal-like vs. luminal subtypes (mean adjusted Rand index 0.14 over 100 random permutations), indicating that the model—and not only the initial mRNA expression profiles of the breast cancer cell lines—was crucial for segregating cell lines by subtype. We further investigated whether the inferred protein activity of breast cancer cell lines—based on the TCGA model alone—correlated with newly available cell line RPPA data from TCPA (Li et al. 2013). For phosphoproteins whose Spearman correlations between measured and inferred activities were above 0.35 on TCGA tumors, we found similarly strong correlations between measured and predicted protein levels on the independent cell line data (Supplemental Fig. S9).

**Figure 3.**
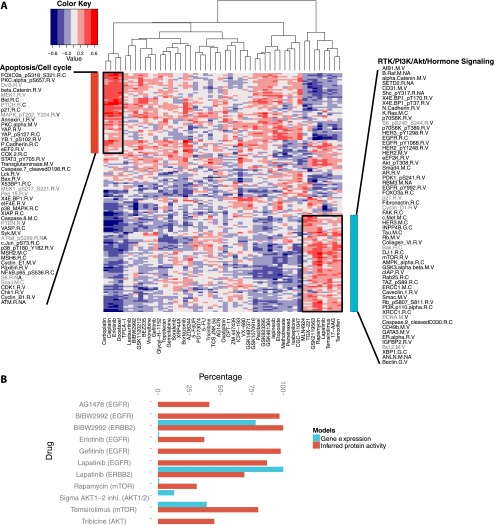
TGCA affinity regression model infers signaling activity in breast cancer cell lines and predicts drug sensitivity. (*A*) Heatmap revealing correlations between inferred protein activities of cell lines (rows) and drug responses (columns). We identified two clusters of drugs from unsupervised analysis: a group consisting mostly of cytotoxic drugs including carboplatin, cisplatin, and docetaxel, but also erlotinib (EGFR); and a group of targeted therapies including tamoxifen (ESR1), 17-AAG (HSP90), temsirolimus (mTOR), rapamycin (mTOR), lapatinib (EGFR, ERBB2), and GSK2119563 (PIK3CA). (*B*) Elastic net drug response models built from inferred protein activity reveal drug targets (shown in parentheses after drug name) more often than models built using gene expression.

To explore possible associations between inferred protein activity and drug response, we first computed Spearman rank correlations between (inferred) protein activity and drug GI50 for each (phospho) protein-drug pair over cell lines. Figure 3A (see also Supplemental Fig. S10) shows the two-way clustering of drugs and proteins by these pairwise Spearman rank correlations; drugs are clustered into groups according to the protein activities that correlate with their response. Several drugs with similar mechanisms of action or affecting a common signaling pathway clustered together. For example, the DNA cross-linking agents carboplatin and cisplatin; the mTOR/PI3K/AKT inhibitors rapamycin, temsirolimus, HSP90, TGX-221, and GSK2119563; as well as the DHFR inhibitors methotrexate and pemetrexed clustered together. Next, to confirm the findings of clustering analysis in a more rigorous way, we also asked, for each pair of drugs, whether ridge regression models trained to predict one drug’s response would generalize to predict the other drug’s response. Results of this transfer learning exercise found similar relationships between drug sensitivities (Supplemental Fig. S11; see Supplemental Methods).

Interestingly, several drugs commonly used in combination for the treatment of breast cancer were often found to cluster together in our analysis. For example, the inferred drug activity of several sets of therapeutics were positively correlated: (1) carboplatin and docetaxel (Chang et al. 2010); (2) tamoxifen (an antineoplastic nonsteroidal selective estrogen receptor modulator) with temsirolimus/rapamycin (mTOR inhibitors) (Baselga et al. 2012) or lapatinib (ERBB2/EGFR inhibitor) (Doss et al. 2012); and (3) HSP90 inhibitors with kinase inhibitors including rapamycin (Francis et al. 2006) and temsirolimus (Okui et al. 2013) (MTOR inhibitors), GSK2119563 (PIK3CA inhibitor), and TGX-221 (PIK3CB). Consistent with the correlation analysis, the combination of tamoxifen with HSP90 inhibitors was found to give better tumor growth inhibition than individual agents in breast cancer cell lines (Giordano et al. 2013). Moreover, lapatinib in combination with rapamycin was shown to be more effective for inhibiting growth of HER2-overexpressing breast cancers resistant to trastuzumab and lapatinib (Gayle et al. 2012). Our results align with several clinical trials demonstrating that inhibiting multiple targets that regulate cancer growth is more effective than monotherapy.

Additionally, sensitivity to carboplatin/cisplatin was associated with the inferred protein activities of CHEK1 and CDK1, which are common markers of triple-negative breast cancer (Fig. 3A; Supplemental Fig. S10B; Heiser et al. 2012). Drugs in this group are associated with proteins that have roles in apoptosis, cell cycle progression, and regulation of cell cycle and immune responses (Fig. 3A; Supplemental Fig. S10B). Conversely, cells with protein activity for luminal- and HER2-associated proteins, such as ESR1 and ERBB2, tend to be resistant to the former group of drugs (carboplatin, cisplatin, docetaxel, erlotinib) but are sensitive to agents targeting PI3K/RTK/ER signaling, autophagy, and differentiation (Fig. 3A; Supplemental Fig. S10C). Moreover, the correlation analysis recovered known drug/target combinations. For example, erlotinib clusters in the former group and has been shown to be effective in a triple-negative xenograft model (Ueno and Zhang 2011). Meanwhile, lapatinib, which clusters in the latter group, is effective for patients with HER2-positive breast cancer and has been shown to synergize with anti-ER therapy (Korkaya et al. 2012; Ithimakin et al. 2013) in a subset of ERBB2-amplified tumors that express ESR1. Indeed, examining the protein activity signatures that correlate with erlotinib and lapatinib, we found that the lapatinib signature includes ERBB2 and EGFR, whereas erlotinib just includes EGFR.

We caution that not all sets of drugs that share similar mechanisms of action (see Supplemental Table S4 for drug targets) or that are used in combination therapies were recovered in this clustering analysis. In particular, relationships between drugs may be missed (1) when the measured drug response does not vary widely across cell lines, or (2) when drugs with similar modes of activity in fact displayed a poorly correlated drug response across cell lines (see Supplemental Figs. S12–S14).

Finally, we trained an elastic net regression model for each drug separately using inferred protein activities as input features and log-transformed GI50 values as output values to learn predictive signatures of drug response. As a baseline comparison method, we also used mRNA expression profiles as input features (see Methods). Use of inferred protein activities as features incurs some loss in prediction accuracy compared to mRNA features (mean fivefold cross-validation MSE error of 0.19 [±0.18] versus 0.18 [±0.14]) (see Methods), perhaps due in part to the difference between tumor and cell line data. However, the drug response signatures associated with inferred protein activities were more likely to include the drug target: For four out of 14 targeted drugs (28%), the mRNA drug signature contained the drug target at least 10 times in 100 iterations of training (see Methods), while for 11 out of 14 targeted drugs (79%), the protein activity drug signatures contained the drug target at least 10% of the time (Fig. 3B). To test for possible selection bias, we then retrained the mRNA models using only the genes in the RPPA list. Again detection of the drug target was less frequent (eight out of 14 drugs, target with positive regression coefficient in at least 10% of models) compared to the inferred protein signatures, suggesting that drug response signatures trained on inferred protein activities may be more interpretable in terms of the mechanism of action of the drug.

### Inferred protein activity of Luminal A cohort predicts survival

Estrogen receptor-positive (ER+) metastatic disease accounts for the majority of breast cancer-related deaths. Luminal A is the most heterogeneous ER+ breast cancer subtype, both molecularly and clinically (Ciriello et al. 2013). Although patients with Luminal A breast cancers have the best survival, the risk of mortality in this subtype persists over decades after the initial diagnosis (Haque et al. 2012). Indeed, Luminal A breast cancers are the only subtype to display a steady drop in survival over a 10-yr period (Haque et al. 2012). Due to clinical significance, we sought to determine whether inferred protein activities based on our model could predict survival in patients with Luminal A breast cancers.

**Figure 4.**
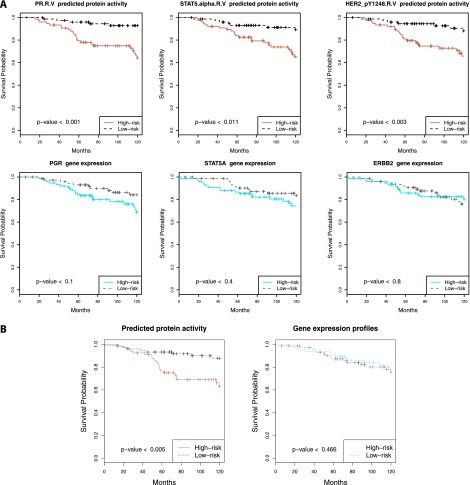
Inferred protein activity predicts survival in patients with Luminal A breast cancers (METABRIC). Using inferred protein activity, a prognostic signature for overall survival was trained on the METABRIC discovery set. Kaplan-Meier survival curves reveal higher- versus lower-risk patients on the validation set using inferred protein activity (*top* panels) but not the corresponding gene expression (*bottom* panels) using (*A*) univariate Cox models for PR, STAT5A, and HER2 and (*B*) multivariate Cox models.

We used the METABRIC cohort (Curtis et al. 2012), which consists of a discovery set and validation set (*n* = 465 and 254 Luminal A tumors, respectively) with mRNA expression profiles and long-term clinical follow-up. First, we used the TCGA-trained affinity regression model to infer protein activity profiles of Luminal A samples in the METABRIC cohort (*Y*^*T*^*DW*). Using the inferred protein activity, we first identified proteins with univariate Cox *P* < 0.001 on the discovery set. Table 3 (Luminal A) and Supplemental Table S5 (Luminal) summarize the univariate survival analysis of significant covariates using predicted protein activities and gene expression profiles. Univariate survival analysis for PGR (Prat et al. 2013) and STAT5A (Peck et al. 2012) associated high protein activity with better overall survival, whereas high ERBB2 and phosphorylated ERBB2 (pY1248) (R Ellsworth, A Valente, and C Shriver, unpubl.) showed a worse prognosis. The association was tested by predicting the risk for each patient in the validation set using the univariate models and performing Kaplan-Meier survival analysis (see Methods). As seen in Figure 4A, univariate models built from inferred protein activity can predict survival in the validation cohort but not models built from the gene expression levels of those proteins. Finally, we built multivariate stepwise Cox regression models using the predicted protein activity and the gene expression profiles of the RPPA proteins on the discovery set (see Methods). Again, in the validation cohort, the model trained with inferred protein activities can predict survival but not the model trained on gene expression profiles corresponding to RPPA-profiled proteins (Fig. 4B). We further confirmed that our multivariate and most of our univariate survival results generalized to Luminal A patients in two other cohorts, TRANSBIG (Supplemental Fig. S15) and NKI (Supplemental Fig. S16).

**Table 3.**
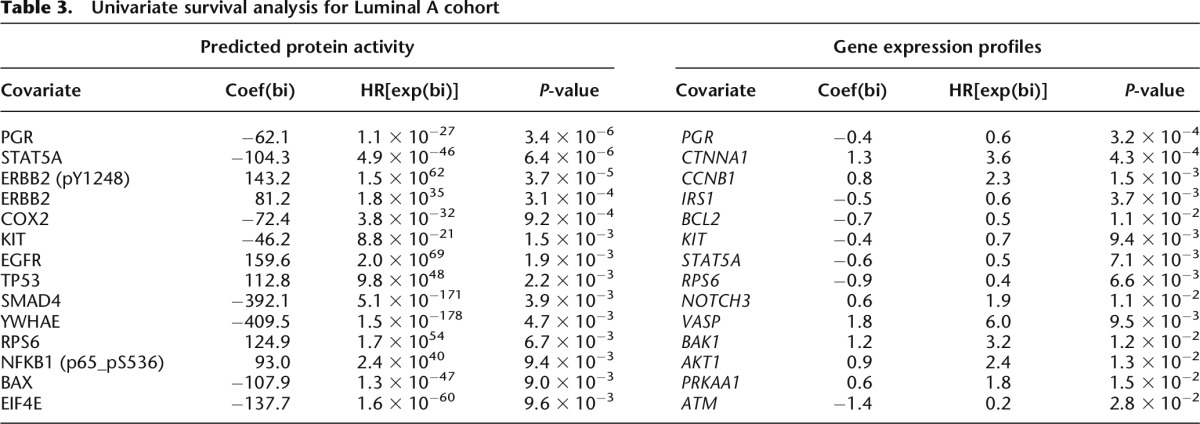
Univariate survival analysis for Luminal A cohort

## Discussion

Deregulation of signaling pathways in cancer results in widespread changes to transcriptional programs. A number of algorithms have been developed to study the dysregulation of gene expression in cancer (Segal et al. 2004; Margolin et al. 2006; Akavia et al. 2010) and to identify post-translational modulators of transcription factor activity from mRNA profiles (Wang et al. 2009). Other methods have tried to infer the activity of signal pathways by integrating mRNA profiles with protein interactions from existing databases, using various graph-theoretic formalisms such as network flow or prize-collecting Steiner trees (Yosef et al. 2009; Vaske et al. 2010; Lan et al. 2011; Tuncbag et al. 2013), and recently the latter approach has been applied to a glioblastoma cell line using both mass spectrometry and expression measurements (Huang et al. 2013). Here we have developed a principled machine learning method to link upstream signaling to downstream transcriptional responses by exploiting the availability of large-scale parallel data from RPPA and mRNA expression arrays from the TCGA breast cancer project. By using a supervised learning approach to weight signaling protein-TF interactions in order to explain the mRNA expression levels of TF target genes, the model implicitly captures the changes in signaling protein activity that are transduced into transcriptional changes. By mapping mRNA expression profiles for new samples through the TF hit matrix and trained interaction model (*Y*^*T*^*DW*), we can infer protein activity profiles from mRNA data. Analysis of the TCGA breast tumor data set showed that (phospho) proteins and TFs that were differentially active in breast cancer subtypes recovered key pathways and downstream effectors that are deregulated in these subtypes.

We further used the TCGA-trained model to infer protein activities from gene expression profiles for breast cancer cell lines for which drug response data was also available. Clustering these inferred protein activity profiles broadly identified two subtypes (basal-like and luminal). Moreover, correlations between inferred protein activities and drug responses in breast cancer cell lines grouped several sets of drugs that are clinically used in combination. This result is consistent with evidence from clinical trials suggesting that therapies that target the same pathway in complementary ways are likely to be effective in combination (Gayle et al. 2012; Boutsikou et al. 2013).

Recently, there have been large-scale efforts to model response to anti-cancer therapies in cell lines with the eventual goal of predicting the clinical efficacy and toxicity of the interrogated drugs. For example, Barretina et al. (2012) used ∼500 fully characterized cell lines from the Cancer Cell Line Encyclopedia (CCLE) along with drug response data from 24 compounds to train models that predict drug sensitivity from cancer cell genotype and mRNA expression levels, representing a step toward the application of predictive models to personalized medicine (Barretina et al. 2012). In an even more ambitious study, the Genomics of Drug Sensitivity in Cancer (GDSC) project (Yang et al. 2013) screened 140 drugs screened against a total of 1200 cancer cell lines and reported both statistical associations between genomic alterations and drug sensitivity as well as regression models. Ultimately, however, regression models of drug response trained on cell line data are unlikely to be used directly as “black box” prediction models in a clinical setting. In our analysis, training drug response models on inferred protein activity profiles led to more interpretable prediction models. Using affinity regression analysis in breast cancer cell line systems with drug response data provides a new strategy for identifying novel drug-signaling pathway associations that can be experimentally validated and potentially translated to clinical trials. Moreover, despite the general problem of inconsistency between large-scale drug response data sets (Haibe-Kains et al. 2013), we found that some of our drug prediction results did indeed generalize (Supplemental Table S6), providing proof-of-principle results in support of our more mechanistically interpretable drug response prediction models.

Although patients with Luminal A breast cancers have the best survival, the risk of mortality in this subtype persists at least over 10 yr after initial diagnosis. Thus, prognostic tests that determine the risk of recurrence are of clinical benefit. Using the Luminal A validation cohort (METABRIC), TRANSBIG and NKI, we found that survival analysis based on inferred protein activities gave superior performance to mRNA expression. Therefore, our approach has prognostic potential and may eventually enable clinicians to choose effective therapies for their patients.

The method we describe has several limitations. Many important TFs bind intronic and intergenic regulatory regions as well as promoters, and the regulatory information at enhancers must ultimately be incorporated into computational models of gene regulation as the field progresses. However, there are significant challenges to incorporating these approaches in the current setting, including the lack of breast tumor DNase-seq data (or other open chromatin/active histone mark data) to reveal the locations of regulatory regions. Moreover, we have a fixed motif representation, where the activity of TFs is inferred by correlation with target expression changes in a linear model; more complex combinatorics of TF binding are not currently modeled. Our method can be used to interpret the effect of mutational/copy number changes in terms of altered TF and signaling protein activities; for example, analysis of tumors that are wild type vs. deleted/mutated for RB1 (Supplemental Fig. S17) or TP53 (Supplemental Fig. S18) produces a candidate list of deregulated TFs/signaling proteins. In future work, we could model the impact of somatic alterations more directly, perhaps by retaining the RPPA representation but including mutation/copy number status as additional covariates.

## Methods

### Data and preprocessing

We downloaded TCGA breast cancer (BRCA) level 3 normalized mRNA expression data derived from the Agilent expression platform and the normalized RPPA protein expression data for 164 proteins and phosphoproteins (Supplemental Material 1, 2) from the Synapse website (https://www.synapse.org/; Derry et al. 2012). Both gene expression and protein expression data were available for 397 BRCA tumors (excluding normal-like). These samples were classified into four main groups using the 50-gene PAM50 model (Parker et al. 2009); 84 basal-like, 48 HER2, 168 LumA, and 97 LumB. We trained our model on equal numbers of samples for each subtype (*n* = 4 × 48).

We further filtered genes whose expression standard deviation was less than 0.65 on a log_2_ scale, resulting in a final set of 4025 genes. Gene expression and protein expression vectors were both mean-centered. Thus, each log-transformed mRNA level was normalized by: 
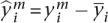
, where 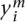 is the expression for gene *i* in the *m*^*th*^ sample, and 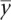 denotes the mean across all the samples. Protein expression levels from the RPPA data were normalized similarly: 
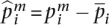
, where 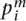 is the expression for protein *i* in the *m*^*th*^ sample, and 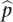 denotes the mean across all the samples.

In order to construct the motif hit matrix, we downloaded the transcription factor (TF) binding site predictions for all target genes (TRANSFAC v7.4) from MSigDB (Liberzon et al. 2011). We removed motifs that have similar sets of targets (Supplemental Material 3). This matrix defines a candidate set of associations between TFs and target genes.

We also downloaded RPPA protein expression data of breast cancer cell lines from TCPA (http://bioinformatics.mdanderson.org/main/TCPA:Overview). We used processed exon array profiling of breast cancer cell lines from Heiser et al. (2012) in ArrayExpress (http://www.ebi.ac.uk/arrayexpress/) under accession number E-MTAB-181. Compound and cell line screening data (preprocessed) were obtained from the published Supplemental Data (Heiser et al. 2012). For CCLE, gene expression and drug information were downloaded from the CCLE website (http://www.broadinstitute.org/ccle). Expression values were log-transformed and mean-centered as described above.

We downloaded the METABRIC (Curtis et al. 2012) from the Synapse website (Derry et al. 2012), TRANSBIG (Desmedt et al. 2007) (from NCBI’s Gene Expression Omnibus [GEO; http://www.ncbi.nlm.nih.gov/geo/] under accession number GSE7390), NKI (van de Vijver et al. 2002) (http://bioinformatics.nki.nl/index.php) gene expression data and survival data. The complete list of data sets used in this study is shown in Supplemental Table S7.

### Inferred transcription factor activity/protein activity and subtype associations

Associations between inferred TF activity and subtype were assessed using the Mann-Whitney *U*-test on inferred activity values over paired groups of samples: (1) basal-like vs. HER2, LumA, LumB; (2) HER2 vs. LumA, LumB; (3) HER2 vs. basal-like; and (4) LumA vs. LumB. To evaluate the significance of each comparison, we used a permutation approach under which 1000 random W (TF-protein interaction) matrices were generated for each TF to compute an empirical null distribution for the test statistic. For each pairwise comparison, we computed the FDR-corrected *P*-value for each TF-subtype association by using the Benjamini-Hochberg procedure on the empirical *P*-values for all tested TFs and identified those that satisfied an FDR threshold of 10%. We first assigned a set of subtypes to TFs based on these *P*-values; then we excluded subtypes whose mean activity had an inconsistent sign compared to the group.

Similarly to the TF-subtype association analysis, we identified subtype-specific signaling proteins by estimating empirical *P*-values relative to randomized versions of the W matrix and reported those passing a 10% FDR threshold.

### Inferred protein activity and drug sensitivity

To analyze how inferred protein activity (*Y*^*T*^*DW*) is related to individual drug response in breast cancer cell lines, we calculated Spearman rank correlations between drug response and inferred activities of individual (phospho) proteins. Protein activity-drug correlations can be either positive or negative. A positive correlation indicates that cell lines that have higher protein activity tend to more be responsive to the tested drug, and a negative correlation indicates that cell lines with high protein activity are more likely resistant to the drug. Further, hierarchical clustering was applied to the protein activity-drug correlation matrix. We also analyzed whether proteins that clustered together interacted with each other using the STRING database (von Mering et al. 2003). For visualization, STRING networks were imported to Cytoscape (Shannon et al. 2003).

Elastic net regression was used to identify associations between inferred protein activity and drug response across breast cancer cell lines. Specifically, inferred protein activities were used as input features to predict log-transformed GI50 values of each drug. Elastic net models were trained with the R package glmnet (Friedman et al. 2010), and fivefold cross-validation was used to optimize the elastic-net mixing parameter α. Potential α values were restricted from 0.001 to 0.2 in order to control the number of final features retained in each run. Under the optimized α, 80% of cell lines across the whole data set were randomly selected to identify biomarkers. The procedure was repeated 100 times for each drug. The final signature of protein activity markers for a drug consisted of all features that appear at least 10 times in any of the 100 runs and whose weights had consistent signs in different signatures. An identical training procedure was used to obtain elastic net drug sensitivity prediction models and signatures from mRNA expression data, where we restricted to the same 4025 genes that we used to train the affinity regression model but also added the drug targets.

### Survival analysis

Cox regression univariate and multivariate analysis was performed using the survival R package (Therneau 1997). Deaths related to other causes were removed from the analysis. Stepwise multivariate model selection based on the Akaike information criterion (AIC) was used to determine the combination of covariates for the multivariate survival models trained on the discovery set. Since inferred protein activities are highly correlated, for multivariate analysis, the procedure was repeated 100 times. For the validation set, using (1) the predicted protein activity profiles and (2) the gene expression profiles corresponding to RPPA proteins, each patient’s risk was calculated, and patients were ranked in descending order. We designated the top 40% of the patients as the high-risk group and the bottom 40% as the low-risk group. The log-rank test was used to compare two Kaplan-Meier survival curves with the null hypothesis that there is no survival difference between the populations.

## Data access

Sample source code and sample data sets are available for download from the Supplemental Material and at http://cbio.mskcc.org/leslielab/affinitybrca/brca-code.zip.
